# Long-term postural control in elite athletes following mild traumatic brain injury

**DOI:** 10.3389/fneur.2022.906594

**Published:** 2022-09-12

**Authors:** Ali Al-Husseini, Anna Gard, Per-Anders Fransson, Yelverton Tegner, Måns Magnusson, Niklas Marklund, Fredrik Tjernström

**Affiliations:** ^1^Department of Clinical Sciences Lund, Neurosurgery, Skåne University Hospital, Lund University, Lund, Sweden; ^2^Department of Clinical Sciences, Lund University, Lund, Sweden; ^3^Department of Health Sciences, Luleå University of Technology, Luleå, Sweden

**Keywords:** mild traumatic brain injury, postural control, sports-related concussion, adaptation, vision, persisting post-concussive symptoms

## Abstract

**Background:**

Traumas to the head and neck are common in sports and often affects otherwise healthy young individuals. Sports-related concussions (SRC), defined as a mild traumatic brain injury (mTBI), may inflict persistent neck and shoulder pain, and headache, but also more complex symptoms, such as imbalance, dizziness, and visual disturbances. These more complex symptoms are difficult to identify with standard health care diagnostic procedures.

**Objective:**

To investigate postural control in a group of former elite athletes with persistent post-concussive symptoms (PPCS) at least 6 months after the incident.

**Method:**

Postural control was examined using posturography during quiet stance and randomized balance perturbations with eyes open and eyes closed. Randomized balance perturbations were used to examine motor learning through sensorimotor adaptation. Force platform recordings were converted to reflect the energy used to maintain balance and spectrally categorized into total energy used, energy used for smooth corrective changes of posture (i.e., <0.1 Hz), and energy used for fast corrective movements to maintain balance (i.e., >0.1 Hz).

**Results:**

The mTBI group included 20 (13 males, mean age 26.6 years) elite athletes with PPCS and the control group included 12 athletes (9 males, mean age 26.4 years) with no history of SRC. The mTBI group used significantly more energy during balance perturbations than controls: +143% total energy, *p* = 0.004; +122% low frequency energy, *p* = 0.007; and +162% high frequency energy, *p* = 0.004. The mTBI subjects also adapted less to the balance perturbations than controls in total (18% mTBI vs. 37% controls, *p* = 0.042), low frequency (24% mTBI vs. 42% controls, *p* = 0.046), and high frequency (6% mTBI vs. 28% controls, *p* = 0.040). The mTBI subjects used significantly more energy during quiet stance than controls: +128% total energy, *p* = 0.034; +136% low-frequency energy, *p* = 0.048; and +109% high-frequency energy, *p* = 0.015.

**Conclusion:**

Athletes with previous mTBI and PPCS used more energy to stand compared to controls during balance perturbations and quiet stance and had diminished sensorimotor adaptation. Sports-related concussions are able to affect postural control and motor learning.

## Introduction

Participation in high-velocity sports, such as football, ice hockey, and rugby, may lead to a risk of sustaining neck and head trauma from a collision with another player, the playing surface, or an advertising board (1). At impact, the athlete may sustain a concussion [termed a sports-related concussion (SRC)], that per definition is a mild traumatic brain injury (mTBI)(2) (2). High-velocity sports carry an increased risk of mTBI (3). The SRC rates are highest in men, but in gender-comparable sports, women have higher concussion rates (3). The reported number of SRCs has increased in the last 20 years, possibly due to increased monitoring and recognition. However, the true incidence is still likely underestimated (4–6).

mTBI may cause persistent neck and shoulder pain, and headache. Typically, these symptoms subside within 14 days of the incident (2, 7–9). However, if the incident is severe, the individual can also experience imbalance, dizziness, and visual disturbances, and symptoms may take months or years to subside (10–12) or remain throughout life (8, 13), termed persistent post-concussive symptoms (PPCS) (14). Recently, we have shown that PPCS athletes suffering from vestibular symptoms commonly have dysfunction of the inferior vestibular nerve (15). As the inferior vestibular nerve conveys information from the posterior semicircular canal and sacculus to the vestibular nucleus, PPCS is likely to diminish the perception of left–right tilt and angular head movements in an anterior–posterior direction. Given the contribution of head position to upright standing, it seems reasonable to assume that PPCS can affect postural control, and particularly when the balance is perturbed.

Imbalance following mTBI is not routinely assessed and individuals can overlook this symptom in a background of pain and headache. When postural control is assessed, it is typically measured from a quiet stance. However, a quiet stance is less sensitive to balance defects than a postural control submitted to balance perturbations. When patients are subjected to increased postural challenges, the likelihood to detect defects is increased and the level of reliance on each sensory system can be assessed. For example, during balance perturbations, the level of reliance on the visual system can be assessed by comparing postural responses with eyes open to eyes closed (16–18). Following an SRC, the results of postural control tests serve a role as supplementary to neurophysiological functioning tests (9, 19, 20), particularly when balance perturbations are used. When balance is perturbed repeatedly, the central nervous system acts to reduce the imbalance. This is a motor learning process called postural adaptation that depends on the integrity of the sensory and motor systems (17, 18). The postural challenge from balance perturbations also increases stress, which may exacerbate balance defects (9, 21). One method of perturbing balance is to apply bilateral mechanical vibration over the gastrocnemii muscles. The vibration produces an increased activation of muscle spindles, and subsequently, an illusion of muscle lengthening, which induces a stretch reflex that creates a posterior displacement. When the vibration ends, corrective mechanisms produce an anterior displacement. When balance perturbations are applied repeatedly, an adaptation in postural control normally occurs, which over time diminishes the amplitude of anterior and posterior displacement (22–24).

The study aimed to determine whether mTBI affects postural stability with eyes open and eyes closed, and the degree of motor learning through sensorimotor adaptation. The reliance on the visual system for postural control was also determined by comparing postural responses with eyes closed to eyes open.

## Materials and methods

### Ethical approval

The investigations were performed in accordance with the latest version of the Helsinki declaration and all subjects gave written informed consent before any assessments. The study was approved by the Ethics Review Board (Dnr 2017/1049), Lund, Sweden.

### Participants

The eligible mTBI group consisted of elite athletes, before their injury active in ice hockey (6 participants), soccer (4), karate (4), handball (2), floorball (2), wrestling (1), and riding (1). They all had to terminate their sports career due to neck and head injuries (SRCs/mTBIs) obtained while executing their sports, which were still causing them various degrees of post-concussive symptoms. They suffered their latest injury more than 6 months before the investigations and reported persistent symptoms for more than 6 months. PPCS is defined as persistent post-concussive symptoms beyond normal clinical recovery, typically more than 3–6 weeks post-injury. All participants in our cohort had symptoms persisting for more than 6 months. The most common complaints included fatigue/low energy (experienced by 100%), headache (in 95%), “pressure in head” (in 95%), difficulty concentrating (in 95%), difficulties in remembering (in 95%), and irritability (in 95%). After medical examinations, including oculomotor and vestibular tests using an Interacoustics™ VNG system (Middelfart, Denmark, version 7.0.9.7), and interviews by a physician, 20 individuals were included in the mTBI group [13 males, mean age 26.6 years (SD 1.2 years, range 19–35 years], with mean weight 73.7 kg (SEM 3.6 kg) and mean height of 178.6 cm (SEM 2.6 cm).

The control group included 12 participants [nine males, mean age 26.4 years (SD 1.6 years, range 20–38 years)], mean weight 71.3 kg (SEM 3.8 kg), mean height 183.2 cm (SEM 2.6 cm) with no history of previous mTBI or neurological/musculoskeletal conditions. The control group was checked by physicians for inclusion/exclusion criteria. Oculomotor and vestibular caloric tests were also done using an Interacoustics™ VNG system (Middelfart, Denmark) to exclude abnormal oculomotor and vestibular performance. The participants were instructed to avoid alcohol at least 48 h before testing.

### Procedure

The participants performed four posturography tests, identically executed by all subjects. Two tests included recording the stability during 120 s of quiet stance while standing with eyes closed (EC) or eyes open (EO) as instructed. Two additional tests, also performed with eyes closed and open, included a 30-s quiet stance period successively followed by a 200 s period with balance perturbations. The balance perturbations were randomized and induced by vibrators strapped over the gastrocnemii (calf) muscles. The vibrators (6 cm long and 1 cm in diameter) produced an 85 Hz and 1.0 mm amplitude vibration. The custom-made vibrators (Section of medical engineering, Skåne University Hospital, Lund, Sweden). The vibration was produced by revolving a 3.5 g weight placed 1.0 mm eccentric on the rotation axis. The vibrators were custom-made for its purpose by the Section of medical engineering, Skåne University Hospital, Lund, Sweden, and the design included using DC motors from Escap, La Chaux-de-Fonds, Switzerland. The vibrations were applied as a sequence of individual balance perturbations by turning ON and OFF the vibrators. The duration of both the ON and OFF state ranged randomly from 0.8 to 6.4 s, according to a pseudorandom binary sequence (PRBS) schedule (25). An identical stimulation sequence was used for all participants and in all balance perturbation tests.

The stability was assessed with a custom-built force platform (Department of Automatic Control, Lund University, Sweden), which recorded both torques and shear forces with six degrees of freedom (d.f.) using force transducers with an accuracy of 0.5 N. A custom-built software (Postcon™, Department of Clinical Sciences, Lund University, Sweden) sampled the force platform data at 50 Hz and produced the balance perturbation sequences by using a 16-bit AD-board (PCI-6036E, National Instruments).

At each posturography test, the subject was instructed to stand in an erect and relaxed posture with their arms folded across the chest. The subject stood barefoot on the force platform and using guidelines, their heels were placed 3 cm apart and their feet placed deviating about 30° open to the front. When performing tests with eyes open, the subject was instructed to focus on a 4 × 6 cm image placed at eye level on a wall 1.5 m in front of them. None of the test subjects had any prior experience with the posturography tests used in the study, and they received no information about how the balance perturbations would affect them. During the posturography tests, the subjects listened to calm classical music using headphones to avoid extraneous sound distractions. The subjects were allowed to rest for 5 min between each of the four posturography tests.

### Analysis

Only the properties of the movements in the anteroposterior direction were analyzed since balance perturbations from calf muscle vibration primarily induce forward and backward movements (24, 26). The variance of the torque values recorded by the force platform was calculated because this parameter corresponds to the energy used toward the support surface to preserve stability (27), i.e., the parameter reflects the efficiency of the central nervous systems' (CNS) control of the standing (28). For a more detailed explanation of the relationship between recorded torque and standing postural control, see Johansson et al. (27).

The force platform recordings were divided into three spectral categories: (a) total torque variance (total energy); (b) torque variance below 0.1 Hz (low-frequency energy); and (c) torque variance above 0.1 Hz (high-frequency energy) using a fifth-order digital Finite Duration Impulse Response (FIR) filter. The filter components were selected to avoid aliasing. This spectral categorization was done to obtain information also about the energy used for corrective changes of posture (i.e., <0.1 Hz) and for fast corrective movements to maintain balance (i.e., >0.1 Hz) (29). Typically, the fast corrective movements (>0.1 Hz) are increased by decreased visual information (29) or by factors like being overweight or fatigued (30). The low-frequency movements (<0.1 Hz) are commonly increased by unstable surface conditions like standing on foam (31, 32).

We analyzed the stability during the two quiet stance tests as one continuous time period (0–120 s). The stability during the two balance perturbation tests was analyzed and segmented into five time periods, a quiet stance period (0–30 s), and during the vibratory stimulation as four consecutive 50-s time periods; Period 1 between 30 and 80 s; Period 2 between 80 and 130 s; Period 3 between 130 and 180 s; and Period 4 between 180 and 230 s. During all the four stimulation periods P1 to P4 analyzed, the vibration stimulus had a similar effective bandwidth in the region of 0.1–2.5 Hz.

### Statistical analysis

Before the statistical analyses were performed, the force platform recordings during the four posturography tests (i.e., the two quiet stance tests and the two balance perturbation tests) were first separated into three spectral bandwidths (total: <0.1 Hz and >0.1 Hz). The torque variance values for these three spectral datasets were thereafter calculated and normalized using the subjects' height and weight to account for anthropometric differences, as these individual factors influence recorded torque values (27). Finally, the normalized torque variances were log-transformed (using natural log) in preparation for the statistical analyses.

As the initial step, statistical analyses were performed with repeated measures of General Linear Model (GLM) Analysis of Variance (ANOVA). The statistical method was used after ensuring that all dataset combinations analyzed in the study fulfilled the three criteria: (1) appropriate independency; (2) produced acceptable sphericity according to the Greenhouse-Geisser evaluation; and (3) that the model residuals had normal or close to a normal distribution, thus validating the method's appropriateness (33–35).

The main factor combinations analyzed for their effects on stability within the three spectral bandwidths (total; <0.1 Hz and >0.1 Hz) during the two balance perturbation tests were:

(1) Main factors Group (Controls vs. mTBI, df 1), Vision (Eyes Open vs. Eyes Closed, df 1), and Repetition (Vibration periods 1–4, df 3). The model parameter Group is a Between-Subjects factor, and the model parameters Vision and Repetition are Within-Subjects variables.
(2) Performed for each individual group: Main factors Vision (Eyes Open vs. Eyes Closed, df 1) and Repetition (Vibration periods 1–4, df 3). The model parameters Vision and Repetition are Within-Subjects variables.

The main factor combinations analyzed for their effects on the stability within the three spectral bandwidths (total; <0.1Hz and > 0.1Hz) during the two quiet stance tests were:

1) Main factors Group (Controls vs. mTBI, df 1) and Vision (Eyes Open vs. Eyes Closed, df 1). The model parameter Group is a Between-Subjects factor, and the model parameter Vision is a Within-Subjects variable.

In all analyses, *p*-values < 0.05 were considered significant. Procedures were utilized to address potential Type I and Type II errors. The need to use Bonferroni correction was considered but regarded as not required as no dataset was included in the Within-subject or the Between-groups tests more than once. The Shapiro-Wilk test revealed that some datasets were not normally distributed, and that normal distribution could not be obtained by log transformation. Thus, non-parametric statistical methods were used in all *post-hoc* statistical evaluations (33).

In *post-hoc* analyses, Within-Subjects paired comparisons were performed to study the effects of Vision and the adaptive changes from Repetition, i.e., the cumulative changes from perturbation period 1 to period 4 (36) using the Wilcoxon matched-pairs signed-rank test (Exact sig. 2-tailed). Between-group comparisons of mTBI vs. controls were performed with Mann-Whitney U Tests (Exact sig. 2 tailed) (33).

## Results

### Stability during the balance perturbation tests

**Figure 1:**
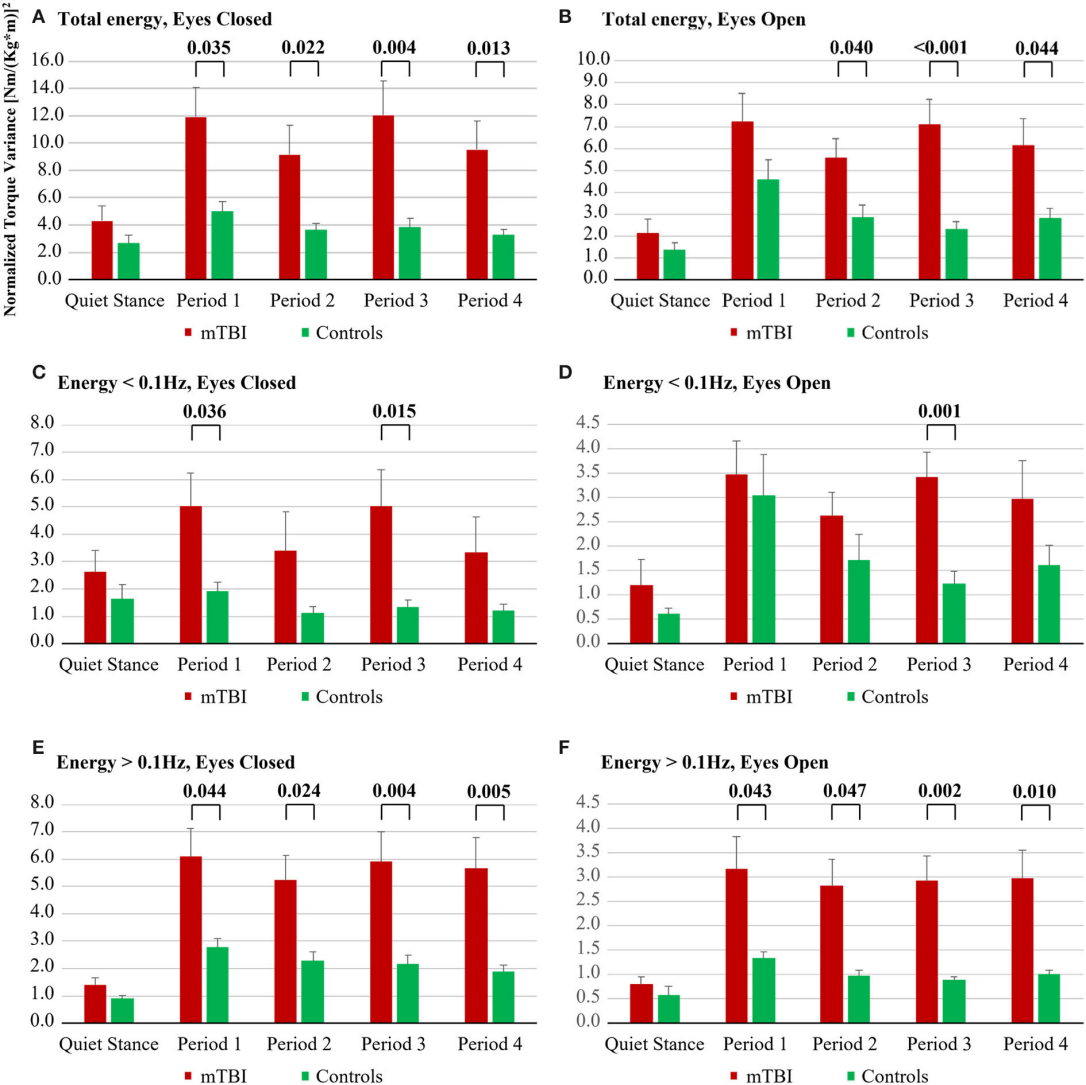
Energy used by mTBI subjects and by controls during the five balance perturbation periods; **(A)** Total energy, Eyes Closed; **(B)** Total energy, Eyes Open; **(C)** Energy < 0.1 Hz, Eyes Closed; **(D)** Energy < 0.1 Hz, Eyes Open; **(E)** Energy > 0.1 Hz, Eyes Closed; **(F)** Energy > 0.1 Hz, Eyes Open.

Repeated measures GLM ANOVA of the model (Group, Vision, Repetition) showed by the main factor Group that subjects who suffered from mTBI used significantly more energy compared with controls during the balance perturbation within all three spectral categories; total (143% more energy, *p* = 0.004), low frequency (122% more, *p* = 0.007), and high frequency (162% more, *p* = 0.004), see Table 1 and Figure 1. The results for the main factor Vision revealed that significantly more energy was used with eyes closed compared with eyes open in the categories: total (51% more, *p* < 0.001) and high frequency (99% more, *p* < 0.001). Analysis of the main factor repetition revealed that across the vibration periods the subjects of both groups used less energy to handle the balance perturbations within all three spectral categories: total (27% less, *p* = 0.001), low frequency (33% less, *p* = 0.012), and high frequency (17% less, *p* = 0.002).

**Table 1:** GLM ANOVA analysis of effects of Group, Vision and Repetition on the stability during balance perturbations.

Energy use within different spectral categories*	**Group**	**Vision**	**Repetition**	**Group × Vision**	**Group × Repetition**	**Vision × Repetition**	**Group × Vision × Repetition**
Total	**0.004 [9.8]**	**<0.001 [28.7]**	**0.001 [12,3]**	0.271 [1.3]	**0.042 [4.5]**	0.322 [1.0]	0.171 [2.0]
<0.1 Hz	**0.007 [8.2]**	0.394 [0.7]	**0.012 [7.1]**	0.198 [1.7]	**0.046 [4.3]**	0.317 [1.0]	0.413 [0.7]
> 0.1 Hz	**0.004 [9.7]**	**<0.001 [136.3]**	**0.002 [12.2]**	0.946 [0.0]	**0.040 [4.6]**	0.143 [2.3]	0.232 [1.5]

*The notation “ < 0.001” means that the *p*-value is smaller than 0.001. *F*-values are presented in the squared parenthesis. The significant differences are marked in bold text.

The Group X Repetition interaction result showed that controls had a better adaptation to the balance perturbations compared with mTBI subjects within all three spectral categories: total (18% mTBI vs. 37% controls, *p* = 0.042), low frequency (24% mTBI vs. 42% controls, *p* = 0.046), and high frequency (6% mTBI vs. 28% controls, *p* = 0.040) bandwidths.

Repeated measures GLM ANOVA analyses of the model (Vision, Repetition) on group level revealed for the main factor Vision that mTBI subjects used significantly more energy with eyes closed compared with eyes open in the spectral categories: total (63% more, *p* < 0.001) and high frequency (93% more, *p* < 0.001), see Table 2 and Figure 1. Analysis of the main factor Repetition revealed that across the vibration periods mTBI subjects used less energy to handle the balance perturbations within all three spectral categories: total (18% less energy, *p* < 0.001), low frequency (24% less, *p* < 0.001), and high frequency (6% less, *p* = 0.009).

**Table 2:** GLM ANOVA analysis of effects of vision and repetition on group level on the stability during balance perturbations.

Energy use within different spectral categories*	**Vision**	**Repetition**	**Vision × Repetition**
mTBI			
Total	**<** **0.001 [39.6]**	**<** **0.001 [30.1]**	0.770 [0.1]
<0.1 Hz	0.701 [0.2]	**<** **0.001 [22.1]**	0.448 [0.6]
> 0.1 Hz	**<** **0.001 [99.2]**	**0.009 [8.6]**	0.605 [0.3]
Controls			
Total	0.051 [4.8]	**0.024 [6.8]**	0.089 [3.5]
<0.1 Hz	0.230 [1.0]	**0.047 [5.0]**	0.399 [0.8]
> 0.1 Hz	**<** **0.001 [47.4]**	**0.002 [17.1]**	0.082 [3.7]

*The notation “ < 0.001” means that the *p*-value is smaller than 0.001. *F*-values are presented in the squared parenthesis. The significant differences are marked in bold text.

Repeated measures GLM ANOVA analyses of the model (Vision, Repetition) on group level revealed for the main factor Vision that control subjects used significantly more energy with eyes closed compared with eyes open in the spectral category; high frequency (116% more, *p* < 0.001), see Table 2 and Figure 1. Analysis of the main factor Repetition revealed that across the vibration periods control subjects used less energy to handle the balance perturbations within all three spectral categories: total (37% less, *p* = 0.024), low frequency (42% less, *p* = 0.047), and high frequency (28% less, *p* = 0.002).

#### Post hoc analysis of group

With eyes closed, during all balance perturbation periods from period 1 to period 4, mTBI subjects used significantly more energy compared with controls in the spectral categories: total (mean 171% more, *p* ≤ 0.035) and high frequency (mean 153% more, *p* ≤ 0.044), see Figure 1. Moreover, during balance perturbation periods 1 and 3, mTBI subjects used significantly more energy than controls in the spectral category: low frequency (mean 210% more, *p* ≤ 0.036).

With eyes open, during balance perturbation periods 2, 3, and 4, the mTBI subject used significantly more energy than controls in the spectral category: total (mean 137% more, *p* ≤ 0.044). During all balance perturbation periods from period 1 to period 4, the mTBI subject used significantly more energy than controls in the spectral category; high frequency (mean 183% more, *p* ≤ 0.047). Moreover, during balance perturbation period 3, the mTBI subject used significantly more energy than controls in the spectral category; low frequency (178% more, *p* < 0.001).

#### Post hoc analysis of vision

The mTBI subjects used significantly more energy with eyes closed compared with eyes open in the spectral category: total—during the initial quiet stance period (103% more, *p* = 0.008) and during all balance perturbation periods from period 1 to period 4 (mean 63% more, *p* ≤ 0.008), see Table 3. The mTBI subjects also used significantly more energy with eyes closed compared with eyes open in the spectral category: high frequency—during the initial quiet stance period (75% more, *p* = 0.019) and during all balance perturbation periods from period 1 to period 4 (mean 93% more, *p* < 0.001). Moreover, mTBI subjects used significantly more energy with eyes closed compared with eyes open in the spectral category: low frequency—during the initial quiet stance period (116% more, *p* = 0.015).

**Table 3:** Differences in stability due to vision for mTBI and control subjects.

**Energy use within different spectral categories**	**Quiet stance**	**Period 1**	**Period 2**	**Period 3**	**Period 4**
mTBI				
Total	**0.008**	**0.002**	**0.006**	**0.002**	**0.001**
<0.1 Hz	**0.015**	0.648	0.985	0.596	0.648
>0.1 Hz	**0.019**	**<** **0.001**	**<** **0.001**	**<** **0.001**	**<** **0.001**
Controls				
Total	**0.009**	0.677	0.064	0.092	0.233
<0.1 Hz	0.151	0.339	0.204	0.850	0.424
>0.1 Hz	**0.003**	**<** **0.001**	**<** **0.001**	**<** **0.001**	**0.005**

The significant differences are marked in bold text.

The control subjects used significantly more energy with eyes closed compared with eyes open in the spectral category: total—during the initial quiet stance period (98% more energy, *p* = 0.009), see Table 3. The control subjects also used significantly more energy with eyes closed compared with eyes open in the spectral category: high frequency—during the initial quiet stance period (54% more, *p* = 0.003) and during all balance perturbation periods from period 1 to period 4 (mean 116% more, *p* ≤ 0.005).

#### Post hoc analysis of adaptation to balance perturbation

In mTBI, the balance perturbations caused a cumulative adaptation of about 34% with eyes closed in the spectral category: low frequency (*p* = 0.048), see Table 4.

**Table 4:** Energy use changes between vibration period 1 and vibration period 4.

Energy use changes*	**Vibration period 1 vs. period 4**	
	**Eyes closed**	**Eyes open**
mTBI		
Total	0.261 (0.80)	0.143 (0.85)
<0.1 Hz	**0.048 (0.66)**	0.452 (0.86)
>0.1 Hz	0.368 (0.93)	0.330 (0.94)
Controls		
Total	**0.016 (0.66)**	0.151 (0.61)
<0.1 Hz	0.233 (0.62)	0.176 (0.53)
>0.1 Hz	**0.002 (0.69)**	0.077 (0.76)

*The quotient value between Period 1 and Period 4 is presented within the parenthesis. A quotient value above 1.0 signifies a worsening performance over time, i.e., that the energy used was higher in Period 4 compared to Period 1. The significant differences are marked in bold text.

In controls, the balance perturbations caused a cumulative adaptation of about 34% on average with eyes closed in the spectral category: total (*p* = 0.016) and a cumulative adaptation of about 31% in the spectral category: high frequency (*p* = 0.002), see Table 4.

### Stability during the quiet stance tests

**Figure 2:**
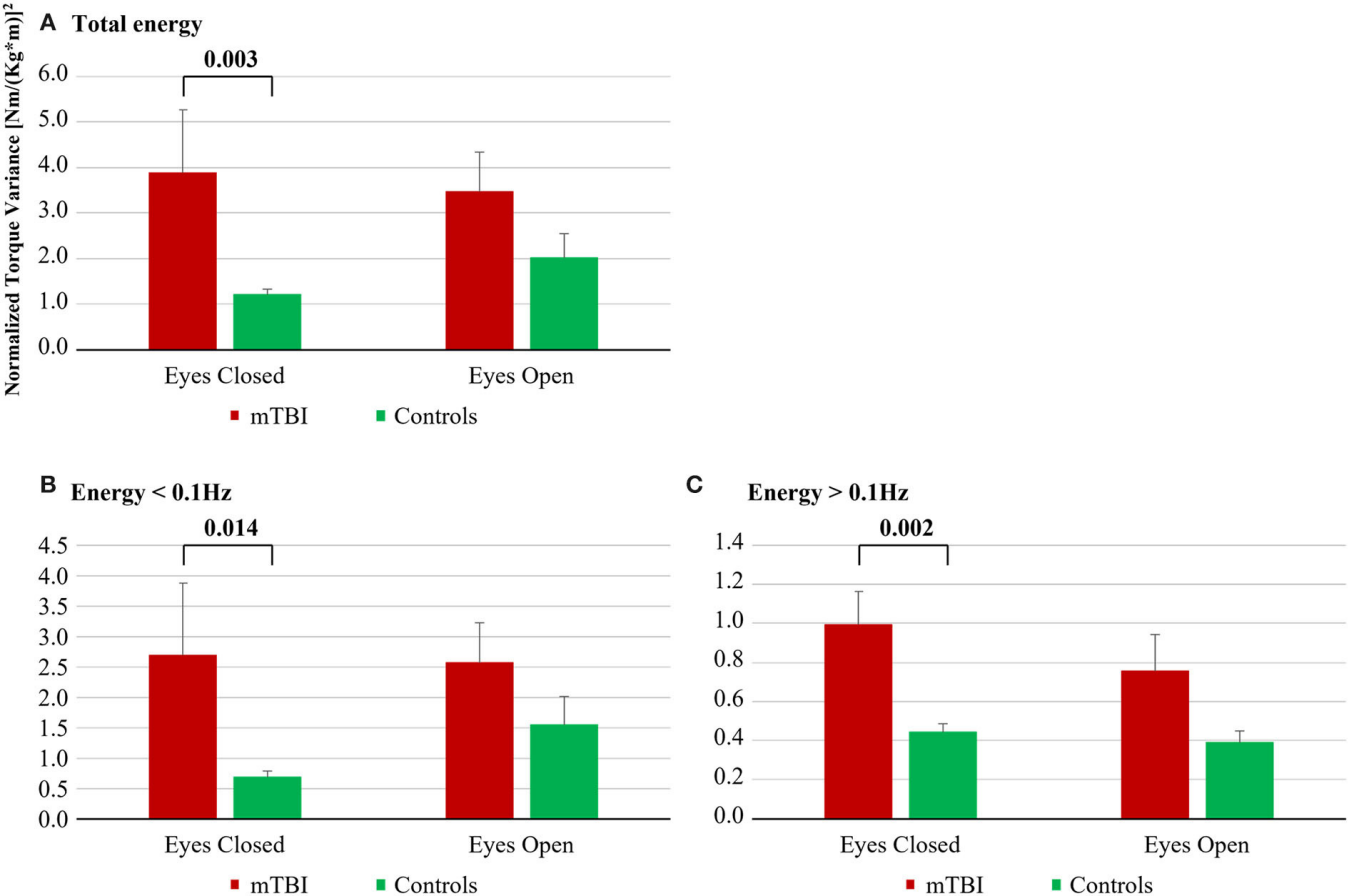
Energy used by mTBI subjects and by controls during the quiet stance tests; **(A)** Total energy; **(B)** Energy < 0.1 Hz; **(C)** Energy > 0.1 Hz.

Repeated measures GLM ANOVA of the model (Group, Vision) showed that mTBI subjects used significantly more energy than controls during quiet stance within all three spectral categories: total (128% more, *p* = 0.034), low frequency (136% more, *p* = 0.048) and high frequency (109% more, *p* = 0.015), see Table 5 and Figure 2. The significant results for the main factor Vision revealed that less energy was used with eyes closed compared with eyes open in the spectral category: low frequency (18% less, *p* = 0.048). Moreover, significantly more energy was used with eyes closed compared with eyes open in the spectral category: high frequency (25% more, *p* = 0.027).

**Table 5:** GLM ANOVA analysis of effects of Group and Vision on the stability during quiet stance.

Energy use within different spectral categories*	**Group**	**Vision**	**Group × Vision**
Total	**0.034 [4.9]**	0.302 [1.1]	0.120 [2.6]
<0.1 Hz	**0.048 [4.3]**	**0.027 [5.4]**	0.254 [1.3]
> 0.1 Hz	**0.015 [6.7]**	**<** **0.001 [14.0]**	0.220 [1.6]

*The notation “ < 0.001” means that the *p*-value is smaller than 0.001. *F*-values are presented in the squared parenthesis. The significant differences are marked in bold text.

#### Post hoc analysis of group

With eyes closed, the mTBI subject used significantly more energy compared with controls in all three spectral categories: total (222% more, *p* = 0.003), low frequency (290% more, *p* = 0.014), and high frequency (124% more, *p* = 0.002), see Figure 2.

## Discussion

The mTBI group used more energy to stand compared to the control group during balance perturbations and quiet stance. They also had poorer sensorimotor adaptation compared to the control group as evidenced by more energy use for repeated balance perturbations. These differences tended to be accentuated when standing with eyes closed and manifested through the higher contribution of fast corrective movements to maintain balance. The findings of this study support the premise that a sports-related concussion might reduce postural control and motor learning through sensorimotor adaptation. An impaired adaptive capacity has both diagnostic and therapeutic implications and might add to the explanation of symptoms perceived in some sufferers of mTBI. A reduced adaptive capacity may equate to prolonged symptoms and reduced effectiveness of rehabilitation. Moreover, our results also indicate the value of assessing postural control in individuals with mTBI.

### General effects of mTBI on stability

The mTBI group used significantly more energy to maintain stability compared with controls during the balance perturbation, both with eyes open and eyes closed, and within all three spectral categories. The spectral categorization was done to obtain detailed information about the energy used for corrective changes of posture and used for fast corrective movements to maintain balance (29). Typically, the fast corrective movements (>0.1 Hz) are increased by decreased visual information (29) or by factors like being overweight or fatigued (30). The slow movements (<0.1 Hz) are commonly increased by unstable surface conditions like standing on foam (31, 32). Intriguingly, the largest differences found between groups were that mTBI subjects used significantly more fast corrective movements than controls. Changes in the fast subconscious movement control, imply that central sensorimotor processes are not operating as efficiently as they should.

The difference in postural control between the mTBI group and the control group was greater during balance perturbations compared to quiet stance. Moreover, with balance perturbations, the mTBI group required more energy to stand with eyes closed and eyes open, whereas quiet stance tests revealed differences between the groups with eyes closed only (Figures 1, 2). Thus, the use of balance perturbations to examine postural control following mTBI adds important information compared to the quiet stance (9, 21).

The predominant cause for persisting symptoms of mTBI is suggested to be central nervous system white matter pathologies, produced by the rotational forces during the trauma (37). However, it is also deemed that all relevant central white matter lesions or disturbances may not be large enough to appear as structural pathologies in conventional neuroimaging (38, 39). In line with this notion, in mTBI patients examined on a median of 22 days after a trauma, a decrease in fractional anisotropy and an increase in mean diffusivity in the cerebellum correlated to imbalance or dizziness symptoms (40). However, Gard et al. (15) recently reported that most of the patients included in this study had impaired saccular and posterior canal functions, in line with a lesion to the inferior vestibular nerves. This was observed without detecting obvious posterior fossa changes on 7 T MRI. Thus, in some mTBI patients, a vestibular dysfunction may contribute to the balance disturbances.

### Adaptation

With repeated balance perturbations, the level of energy used to maintain upright standing diminished in the control group as expected, showing sensorimotor adaptation. However, the degree of sensorimotor adaptation was diminished in the mTBI group within all three spectral categories. When balance is perturbed, the brain detects the imbalance and makes changes to body posture, and generates predictive muscle responses to reduce the imbalance when the perturbation is repeated (24, 41–43). This adaptive response is mediated initially by cortical mechanisms and thereafter by subcortical mechanisms (44, 45). The reduced ability to adapt to repeated perturbances suggests a reduction of such a function. Sensorimotor adaptation is crucial to sporting performance and therefore, a reduced ability in this respect will affect competitiveness at the highest levels. However, sensorimotor adaptation is also crucial to everyday motor activities including postural and locomotor control.

Another possibility to be contemplated is that mTBI subjects might distrust their postural ability and react to this with poor adaptation in the same way as patients with a fear of falling or persistent postural perceptual dizziness (PPPD) do (46–48). However, our mTBI subjects were elite athletes, and a fear of falling seemed less likely as the mere mechanism of the observations. Moreover, a common reaction to a psychologically instigated fear of falling is increased recorded activity within the >0.1 Hz frequency range but normal or lower than normal activity in the <0.1 Hz frequency range, due to increased rigidity caused by co-contraction of the anti-gravity muscles (49). The mTBI subjects did not display such characteristics but presented significantly higher activity than controls both in the <0.1 Hz and in the >0.1 Hz frequency ranges both during quiet stance and balance perturbations.

### Role of vision

The differences between mTBI and controls were accentuated when standing with eyes closed. With visual feedback, the mTBI subjects presented smaller differences from controls in both adaptations and posture control measurements. That is, the mTBI subjects had less need for fast corrective movements and less need for major changes of posture. Arguably, the frame of reference provided by vision helps to stabilize posture and reduce the effects of perturbations. An alternative explanation is that the subjects with mTBI become more dependent on visual information for balance control. The latter is in line with the finding that most of these subjects showed signs of vestibular dysfunction (15). Symptoms that might be of vestibular origin following SRC are commonly reported. In one study, 68% of athletes reported dizziness and 36% imbalance following SRC (50). Most of the symptoms resolved within 2–7 days (67.1 %), but in a small group (7%) it persisted for more than a month (50). Of note, the mTBI subjects assessed in the current study suffered their injury more than 6 months before the study and reported persistent symptoms for more than 6 months. Thus, at the time of this study, all mTBI subjects should have recovered from the typical SRC symptoms of head and neck pain and of headache. Moreover, any vestibular impairment from the mTBI incident should have been compensated for by upweighting visual and somatosensory information and suppressing less accurate vestibular information.

When changing from standing with eyes open to standing with eyes closed, there is a shift from using vision as the predominant information source for stability control to instead using the mechanoreceptive and proprioceptive systems as the predominant information source. Vision provides a frame of reference for the head movements, and thus, for the movements of the top segment in our biomechanically multisegmented body. This promotes using a segmental up-down postural control strategy. The predominant frame of reference for the mechanoreceptive and proprioceptive systems is the lowest body segment's contact with the ground, which promotes the use of a segmental bottom-up postural control strategy. This change in control mode and available reliable sensory information is reflected by increased use of fast corrective movements, as the mechanoreceptive and proprioceptive sensory systems are able to support such feedback control processes (29). Both the mTBI athletes and controls changed in a similar way to predominantly using a mechanoreceptive/proprioceptive control mode. However, the mTBI subjects used significantly more energy with their eyes closed, not only within the high-frequency range but also within the total frequency range.

Maintaining postural control is dependent upon accurate sensory cues. The sensory information from vision, vestibular, and somatosensory receptors are conveyed to the central nervous system to be integrated into an aggregative perception of the present body position and movements. Thus, there can be several potential reasons why stability is poorer with eyes closed. This includes the possibility of diminished somatosensory information, reduced integration of somatosensory information, and an impaired exaction of postural responses in a mechanoreceptive/proprioceptive control mode. Postural instability may follow a vestibular nerve lesion following an mTBI, particularly acutely, and with eyes closed. To compensate for this, the central nervous system may become increasingly dependent upon visual information (51). However, postural control was significantly poorer in mTBI subjects than in controls with eyes open as well, evidenced by the use of more energy to stand, and this difference tended to be accentuated during the latter periods of the balance perturbation. Noteworthy, the level of fast corrective movements in vision control mode was as high in mTBI subjects as it was in controls in mechanoreceptive/proprioceptive control mode. Hence, mTBI subjects were able to utilize vision to enhance their stability, but access to visual information was not alone able to compensate for the deficits in postural control in mTBI subjects.

### Limitations

We evaluated a group of mTBI athletes under a specific set of criteria, e.g., that they suffered from significant and long-term (>6 months) post-concussive/mTBI symptoms. However, our selected cohort is likely not representative of all SRC athletes, and the findings of our study may not be observed in athletes with a more limited duration of symptoms. The results are also based on a relatively small sample size, and the study may only apply to mTBI subjects with a similar medical history. Another limitation of the study is that we included no control group of asymptomatic SRC athletes. We are also aware that additional relevant information might have been obtained if the subjects had been assessed earlier after subjects sustaining their injury and if the subjects had been assessed repeatedly to monitor recovery. The included mTBI subjects were young adults who exercised more than the average population. The controls were matched in terms of age and physical activity but were not elite level.

## Conclusion

The mTBI subjects had significantly poorer stability than controls during balance perturbations and quiet stance. Moreover, controls had better adaptation to balance perturbations than mTBI subjects. These differences tended to be accentuated when standing with eyes closed and were manifested by an increased contribution of fast corrective movements to maintain balance. Hence, the findings of this study support the premise that sports-related concussions might affect postural control and sensorimotor adaptation. Furthermore, the findings suggest that posturography could be included in the battery of diagnostic tests to assess mTBI. Results from posturography may predict the length of expected recovery and the outcome of rehabilitation since sensorimotor adaptation is key to both.

## Data availability statement

The raw data supporting the conclusions of this article will be made available by the authors, without undue reservation.

## Ethics statement

The studies involving human participants were reviewed and approved by Ethics Review Board (Dnr 2017/1049), Lund University, Sweden. The patients/participants provided their written informed consent to participate in this study.

## Author contributions

MM, NM, FT, and YT contributed to the conception and design of the study. AA-H and AG carried out the data collection. P-AF performed the statistical analysis. MM, P-AF, FT, AA-H, and AG interpreted the results. AA-H, AG, P-AF, and MM wrote the first draft of the manuscript. All authors contributed to manuscript revision, read, and approved the submitted version.

## Funding

This work was funded by the Swedish Research Council for Sport Science CIF 2021-0105, Swedish Brain Foundation 2020, Swedish Research Council VR 2018-02500 (all to NM), and hospital ALF funds (to NM and MM).

## Conflict of interest

The authors declare that the research was conducted in the absence of any commercial or financial relationships that could be construed as a potential conflict of interest.

## Publisher's note

All claims expressed in this article are solely those of the authors and do not necessarily represent those of their affiliated organizations, or those of the publisher, the editors and the reviewers. Any product that may be evaluated in this article, or claim that may be made by its manufacturer, is not guaranteed or endorsed by the publisher.

## References

[1] Prien A Grafe A Rossler R Junge A Verhagen E. Epidemiology of head injuries focusing on concussions in team contact sports: a systematic review. Sports Med. (2018) 48:953–69. 10.1007/s40279-017-0854-4 29349651

[2] McCrory P Feddermann-Demont N Dvorak J Cassidy JD McIntosh A Vos PE. What is the definition of sports-related concussion: a systematic review. Br J Sports Med. (2017) 51:877–87. 10.1136/bjsports-2016-097393 29098981

[3] Pierpoint LA Collins C. Epidemiology of Sport-Related Concussion. Clin Sports Med. (2021) 40:1–18. 10.1016/j.csm.2020.08.013 33187601

[4] Ramkumar PN Navarro SM Haeberle HS Luu BC Jang A Frangiamore SJ. Concussion in American versus european professional soccer: a decade-long comparative analysis of incidence, return to play, performance, and longevity. Am J Sports Med. (2019) 47:2287–93. 10.1177/0363546519859542 31303010

[5] Patel BH Okoroha KR Jildeh TR Lu Y Idarraga AJ Nwachukwu BU. Concussions in the national basketball association: analysis of incidence, return to play, and performance from 1999 to 2018. Orthop J Sports Med. (2019) 7:2325967119854199. 10.1177/2325967119854199 31276004 PMC6598335

[6] Pauelsen M Nyberg G Tegner C Tegner Y. Concussion in ice hockey-a cohort study across 29 seasons. Clin J Sport Med. (2017) 27:283–7. 10.1097/JSM.0000000000000347 28449005

[7] McCrea M Guskiewicz KM Marshall SW Barr W Randolph C Cantu RC. Acute effects and recovery time following concussion in collegiate football players: the NCAA concussion study. JAMA. (2003) 290:2556–63. 10.1001/jama.290.19.2556 14625332

[8] McCrea M Guskiewicz K Randolph C Barr WB Hammeke TA Marshall SW. Incidence, clinical course, and predictors of prolonged recovery time following sport-related concussion in high school and college athletes. J Int Neuropsychol Soc. (2013) 19:22–33. 10.1017/S1355617712000872 23058235

[9] McCrory P Meeuwisse WH Aubry M Cantu B Dvorak J Echemendia RJ. Consensus statement on concussion in sport: the 4th International Conference on Concussion in Sport held in Zurich, November 2012. Br J Sports Med. (2013) 47:250–8. 10.1136/bjsports-2013-092313 23479479

[10] Lau BC Kontos AP Collins MW Mucha A Lovell MR. Which on-field signs/symptoms predict protracted recovery from sport-related concussion among high school football players? Am J Sports Med. (2011) 39:2311–8. 10.1177/0363546511410655 21712482

[11] Anzalone AJ Blueitt D Case T McGuffin T Pollard K Garrison JC. A positive vestibular/ocular motor screening (VOMS) is associated with increased recovery time after sports-related concussion in youth and adolescent athletes. Am J Sports Med. (2017) 45:474–9. 10.1177/0363546516668624 27789472

[12] Sinnott AM Elbin RJ Collins MW Reeves VL Holland CL Kontos AP. Persistent vestibular-ocular impairment following concussion in adolescents. J Sci Med Sport. (2019) 22:1292–7. 10.1016/j.jsams.2019.08.004 31521485 PMC6825555

[13] Hiploylee C Dufort PA Davis HS Wennberg RA Tartaglia MC Mikulis D. Longitudinal study of postconcussion syndrome: not everyone recovers. J Neurotrauma. (2017) 34:1511–23. 10.1089/neu.2016.4677 27784191 PMC5397249

[14] Tator CH Davis HS Dufort PA Tartaglia MC Davis KD Ebraheem A. Postconcussion syndrome: demographics and predictors in 221 patients. J Neurosurg. (2016) 125:1206–16. 10.3171/2015.6.JNS15664 26918481

[15] Gard A Al-Husseini A Kornaropoulos EN De Maio A Tegner Y Bjorkman-Burtscher I. Post-concussive vestibular dysfunction is related to injury to the inferior vestibular nerve. J Neurotrauma. (2022) 39:829–40. 10.1089/neu.2021.0447 35171721 PMC9225415

[16] Bzduskova D Valkovic P Hirjakova Z Kimijanova J Hlavacka F. Parkinson's disease versus ageing: different postural responses to soleus muscle vibration. Gait Posture. (2018) 65:169–75. 10.1016/j.gaitpost.2018.07.162 30558926

[17] Patel M Fransson PA Magnusson M. Effects of ageing on adaptation during vibratory stimulation of the calf and neck muscles. Gerontology. (2009) 55:82–91. 10.1159/000188114 19096202

[18] Einarsson EJ Patel M Petersen H Wiebe T Fransson PA Magnusson M. Decreased postural control in adult survivors of childhood cancer treated with chemotherapy. Sci Rep. (2016) 6:36784. 10.1038/srep36784 27830766 PMC5103202

[19] Guskiewicz KM. Assessment of postural stability following sport-related concussion. Curr Sports Med Rep. (2003) 2:24–30. 10.1249/00149619-200302000-00006 12831673

[20] Guskiewicz KM Ross SE Marshall SW. Postural stability and neuropsychological deficits after concussion in collegiate athletes. J Athl Train. (2001) 36:263–73. 12937495 PMC155417

[21] Temme LA St Onge P Bleiberg J. A History of mild traumatic brain injury affects peripheral pulse oximetry during normobaric hypoxia. Front Neurol. (2016) 7:149. 10.3389/fneur.2016.00149 27708611 PMC5030829

[22] Tjernstrom F Fransson PA Hafstrom A Magnusson M. Adaptation of postural control to perturbations–a process that initiates long-term motor memory. Gait Posture. (2002) 15:75–82. 10.1016/S0966-6362(01)00175-8 11809583

[23] Patel M Gomez S Lush D Fransson PA. Adaptation and vision change the relationship between muscle activity of the lower limbs and body movement during human balance perturbations. Clin Neurophysiol. (2009) 120:601–9. 10.1016/j.clinph.2008.11.026 19136294

[24] Fransson PA Hafstrom A Karlberg M Magnusson M Tjader A Johansson R. Postural control adaptation during galvanic vestibular and vibratory proprioceptive stimulation. IEEE Trans Biomed Eng. (2003) 50:1310–9. 10.1109/TBME.2003.819851 14656060

[25] Johansson R Magnusson M. Human postural dynamics. Crit Rev Biomed Eng. (1991) 18:413–37. 1855384

[26] Ivanenko YP Talis VL Kazennikov OV. Support stability influences postural responses to muscle vibration in humans. Eur J Neurosci. (1999) 11:647–54. 10.1046/j.1460-9568.1999.00471.x 10051765

[27] Johansson R Fransson PA Magnusson M. Optimal coordination and control of posture and movements. J Physiol Paris. (2009) 103:159–77. 10.1016/j.jphysparis.2009.08.013 19671443

[28] Riccio GE Stoffregen TA. Affordances as constraints on the control of stance. Hum Mov Sci. (1988) 7:265–300. 10.1016/0167-9457(88)90014-0

[29] Kristinsdottir EK Fransson PA Magnusson M. Changes in postural control in healthy elderly subjects are related to vibration sensation, vision and vestibular asymmetry. Acta Otolaryngol. (2001) 121:700–6. 10.1080/00016480152583647 11678169

[30] Ledin T Fransson PA Magnusson M. Effects of postural disturbances with fatigued triceps surae muscles or with 20% additional body weight. Gait Posture. (2004) 19:184–93. 10.1016/S0966-6362(03)00061-4 15013507

[31] Patel M Fransson PA Johansson R Magnusson M. Foam posturography: standing on foam is not equivalent to standing with decreased rapidly adapting mechanoreceptive sensation. Exp Brain Res. (2011) 208:519–27. 10.1007/s00221-010-2498-6 21120458

[32] Fransson PA Gomez S Patel M Johansson L. Changes in multi-segmented body movements and EMG activity while standing on firm and foam support surfaces. Eur J Appl Physiol. (2007) 101:81–9. 10.1007/s00421-007-0476-x 17503068

[33] Altman D. Practical Statistics for Medical Research. New York, NY: Chapman & Hall. (1991). 10.1201/9780429258589

[34] Vogt WP. Dictionary of Statistics and Methodology: A Non-Technical Guide for the Social Sciences. Thousand Oaks, CA: Sage Publications. (1999).

[35] van den Berg RG. SPSS Repeated Measures ANOVA Tutorial. Available online at: https://www.spss-tutorials.com/spss-repeated-measures-anova/comment-page-1/#comments2015, https://www.spss-tutorials.com/spss-repeated-measures-anova/comment-page-1/#comments.

[36] Patel M Gomez S Berg S Almbladh P Lindblad J Petersen H. Effects of 24-h and 36-h sleep deprivation on human postural control and adaptation. Exp Brain Res. (2008) 185:165–73. 10.1007/s00221-007-1143-5 17932662

[37] McKee AC Daneshvar DH Alvarez VE Stein TD. The neuropathology of sport. Acta Neuropathol. (2014) 127:29–51. 10.1007/s00401-013-1230-6 24366527 PMC4255282

[38] Gardner A Kay-Lambkin F Stanwell P Donnelly J Williams WH Hiles A. A systematic review of diffusion tensor imaging findings in sports-related concussion. J Neurotrauma. (2012) 29:2521–38. 10.1089/neu.2012.2628 22950876

[39] Chamard E Lichtenstein JD. A systematic review of neuroimaging findings in children and adolescents with sports-related concussion. Brain Inj. (2018) 32:816–31. 10.1080/02699052.2018.1463106 29648462

[40] Alhilali LM Yaeger K Collins M Fakhran S. Detection of central white matter injury underlying vestibulopathy after mild traumatic brain injury. Radiology. (2014) 272:224–32. 10.1148/radiol.14132670 24735411

[41] Fransson PA Kristinsdottir EK Hafstrom A Magnusson M Johansson R. Balance control and adaptation during vibratory perturbations in middle-aged and elderly humans. Eur J Appl Physiol. (2004) 91:595–603. 10.1007/s00421-003-1013-1 14985989

[42] Fransson PA Tjernstrom F Hafstrom A Magnusson M Johansson R. Analysis of short- and long-term effects of adaptation in human postural control. Biol Cybern. (2002) 86:355–65. 10.1007/s00422-001-0305-y 11984650

[43] Fransson PA Johansson R Tjernstrom F Magnusson M. Adaptation to vibratory perturbations in postural control. IEEE Eng Med Biol Mag. (2003) 22:53–7. 10.1109/MEMB.2003.1195696 12733459

[44] Honeine JL Crisafulli O Schieppati M. Body sway adaptation to addition but not withdrawal of stabilizing visual information is delayed by a concurrent cognitive task. J Neurophysiol. (2017) 117:777–85. 10.1152/jn.00725.2016 27903641 PMC5310235

[45] Patel M Kaski D Bronstein AM. Attention modulates adaptive motor learning in the 'broken escalator' paradigm. Exp Brain Res. (2014) 232:2349–57. 10.1007/s00221-014-3931-z 24715102

[46] Tjernstrom F Fransson PA Holmberg J Karlberg M Magnusson M. Decreased postural adaptation in patients with phobic postural vertigo–an effect of an “anxious” control of posture? Neurosci Lett. (2009) 454:198–202. 10.1016/j.neulet.2009.03.020 19429083

[47] Wuehr M Pradhan C Novozhilov S Krafczyk S Brandt T Jahn K. Inadequate interaction between open- and closed-loop postural control in phobic postural vertigo. J Neurol. (2013) 260:1314–23. 10.1007/s00415-012-6797-7 23263595

[48] Staab JP Eckhardt-Henn A Horii A Jacob R Strupp M Brandt T. Diagnostic criteria for persistent postural-perceptual dizziness (PPPD): consensus document of the committee for the classification of vestibular disorders of the barany society. J Vestib Res. (2017) 27:191–208. 10.3233/VES-170622 29036855 PMC9249299

[49] Holmberg J Karlberg M Fransson PA Magnusson M. Phobic postural vertigo: body sway during vibratory proprioceptive stimulation. Neuroreport. (2003) 14:1007–11. 10.1097/00001756-200305230-00020 12802192

[50] Chorney SR Suryadevara AC Nicholas BD. Audiovestibular symptoms as predictors of prolonged sports-related concussion among NCAA athletes. Laryngoscope. (2017) 127:2850–3. 10.1002/lary.26564 28349568

[51] Bronstein AM Hood JD Gresty MA Panagi C. Visual control of balance in cerebellar and parkinsonian syndromes. Brain. (1990) 113:767–79. 10.1093/brain/113.3.767 2364268

